# Social Network Analysis Reveals Potential Fission-Fusion Behavior in a Shark

**DOI:** 10.1038/srep34087

**Published:** 2016-09-30

**Authors:** Danielle E. Haulsee, Dewayne A. Fox, Matthew W. Breece, Lori M. Brown, Jeff Kneebone, Gregory B. Skomal, Matthew J. Oliver

**Affiliations:** 1 College of Earth, Ocean, and Environment, University of Delaware, Lewes, DE, 19958, USA; 2 Department of Natural Resources, Delaware State University, Dover, DE, 19901, USA; 3 Anderson Cabot Center for Ocean Life, New England Aquarium, Boston, MA, 02110, USA; 4 Massachusetts Division of Marine Fisheries, New Bedford, MA, 02740, USA

## Abstract

Complex social networks and behaviors are difficult to observe for free-living marine species, especially those that move great distances. Using implanted acoustic transceivers to study the inter- and intraspecific interactions of sand tiger sharks *Carcharias taurus*, we observed group behavior that has historically been associated with higher order mammals. We found evidence strongly suggestive of fission-fusion behavior, or changes in group size and composition of sand tigers, related to five behavioral modes (summering, south migration, community bottleneck, dispersal, north migration). Our study shows sexually dimorphic behavior during migration, in addition to presenting evidence of a potential solitary phase for these typically gregarious sharks. Sand tigers spent up to 95 consecutive and 335 cumulative hours together, with the strongest relationships occurring between males. Species that exhibit fission-fusion group dynamics pose a particularly challenging issue for conservation and management because changes in group size and composition affect population estimates and amplify anthropogenic impacts.

While many sharks are solitary predators, some are known to live in groups and are suspected of engaging in complex social behaviors^1^, others simply aggregate due to similar habitat, food, or mating requirements. Evidence of complex social behaviors in sharks and other elasmobranchs is sparse, however we are beginning to understand the importance of studying shark aggregations. Many studies have considered the costs and benefits of group living in terrestrial and aquatic systems, but understanding how and why animals form groups remains a challenge^2^. It is widely accepted that when costs of fusion (e.g., intraspecific competition for food and mates, exposure to diseases) outweigh the benefits (e.g., defense from predators, information sharing, mating opportunities), large groups fission and form smaller sub-groups to maximize fitness in different environmental and intraspecific group settings. Studies have observed fission-fusion behavior in response to changes in prey availability^3^,^4^,^5^, risk of predation^6^,^7^, and territorial competition^8^, but this behavior has been primarily documented in mammals, including humans^9^, non-human primates^3^,^8^,^10^, bats^11^,^12^, and cetaceans^4^,^5^, in addition to reptile^13^,^14^ and fish species^15^, but not in elasmobranchs. Here we present an innovative analysis of the intra- and interspecific group dynamics observed for the sand tiger shark *Carcharias taurus* during its annual migration.

Sand tigers have high brain to body mass ratios when compared to other *Chondrichthyes*^16^, and therefore may have the ability to maintain complex social structures and social behaviors^17^,^18^ such as coordinated group feeding behaviors^19^ similar to those observed in marine mammals^20^. In addition, sand tigers are a vulnerable species due their inherently low fecundity (producing 1–2 pups every other year) and historic overfishing^21^. Here we provide strong evidence for fission-fusion dynamics in an elasmobranch and explain those dynamics in relation to sand tigers’ annual migration along the East Coast of the USA. Understanding changes in the composition and size of sand tiger groups may be useful for effective conservation and management strategies because these behavioral dynamics allow perturbations to disproportionately affect different life history stages.

## Results and Discussion

**Figure 1:**
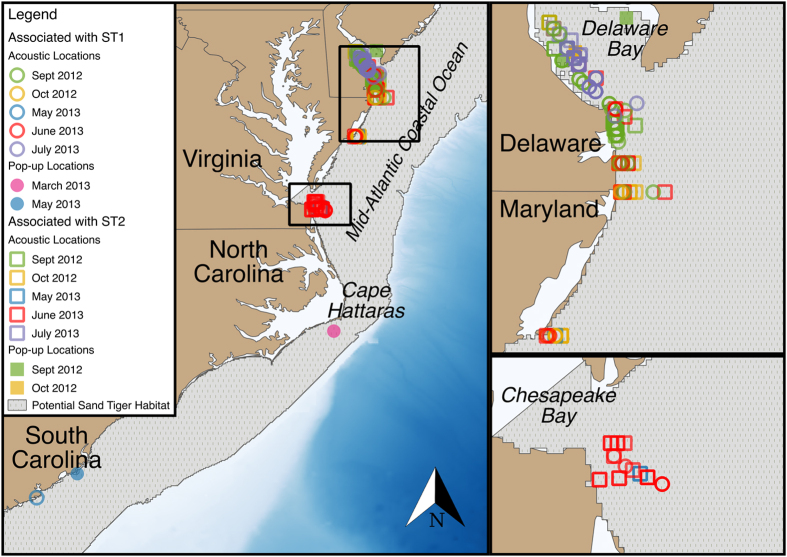
Map of acoustic detection locations and Pop-up Satellite Arcival Tag pop-up locations associated with sand tigers *Carcharias taurus* ST1 and ST2 from September 2012 to July 2013 along the East Coast, USA. Locations represent primary, secondary and tertiary locations (see Supplemental Material and Methods for details). Potential sand tiger habitat represents waters <200 m^40^. Map was created using QGIS version 2.8.3 (www.qgis.org), and includes bathymetry data from Amante and Eakins^48^.

Two VEMCO Mobile Transceivers (VMTs) implanted in sand tigers, hereafter referred to as ST1 and ST2, were successfully recovered after approximately one year (336 and 352 days respectively) at liberty (see Methods for details)^22^. VMTs are unique in that they are able to transmit coded acoustic pings unique to the animal carrying the tag, as well as receive and archive coded acoustic pings from compatible transmitters nearby. Therefore, VMTs are capable of recording thousands of detections from telemetered animals in the ocean. ST1 and ST2 were implanted with the VMTs on the 24 August 2012, and were recaptured within 16 days of each other in 2013 (26 July and 11 August respectively) and 9 km apart in the Delaware Bay. These sand tigers were also tagged with Pop-up Satellite Archival Tags (PSATs) to potentially add a spatial context to detection events, however little useable spatial information was recovered from the PSATs deployed (see Methods for details). Both individuals were considered mature males (194 and 198 cm fork lengths, respectively) at time of tagging, based on published size at maturity data for this species^23^. Documented locations of ST1 and ST2 from moored acoustic receivers and the pop-up locations of the PSATs (Fig. 1) revealed the typical coastal migration patterns for this species^24^,^25^. Range testing studies of VMTs mounted on animals and underwater vehicles show a detection range of ~400 m^26^, but that range may be reduced in a VMT implanted in an animal. This suggests that detection events represent an encounter of two tagged animals within a few hundred meters of each other. ST1 recorded 29,646 detection events from seven species of fish carrying compatible VEMCO acoustic transmitters (69 kHz coded); ST2 recorded 44,210 detection events from five fish species (Table 1). ST1 and ST2 detected 52.3% and 61.5% respectively of telemetered sand tigers in the region (Table 1). Rarefaction curves for the detection records of ST1 and ST2 suggest that the tagged sand tiger population was well sampled (Supplemental Fig. 1). An intra- and interspecific Bray-Curtis similarity analysis revealed monthly changes in the group composition encountered by ST1 and ST2 throughout their annual migration (Supplemental Fig. 2), and will be discussed in detail below. In addition, we used Shannon’s Index to quantify the diversity (individual richness and evenness) of sand tigers encountered throughout each month (Supplemental Fig. 3). From these community changes, five general behavioral modes were inferred to assist in interpretation of the fission-fusion dynamics: summering, south migration, community bottleneck, dispersal, and north migration.

### Summering

**Figure 2:**
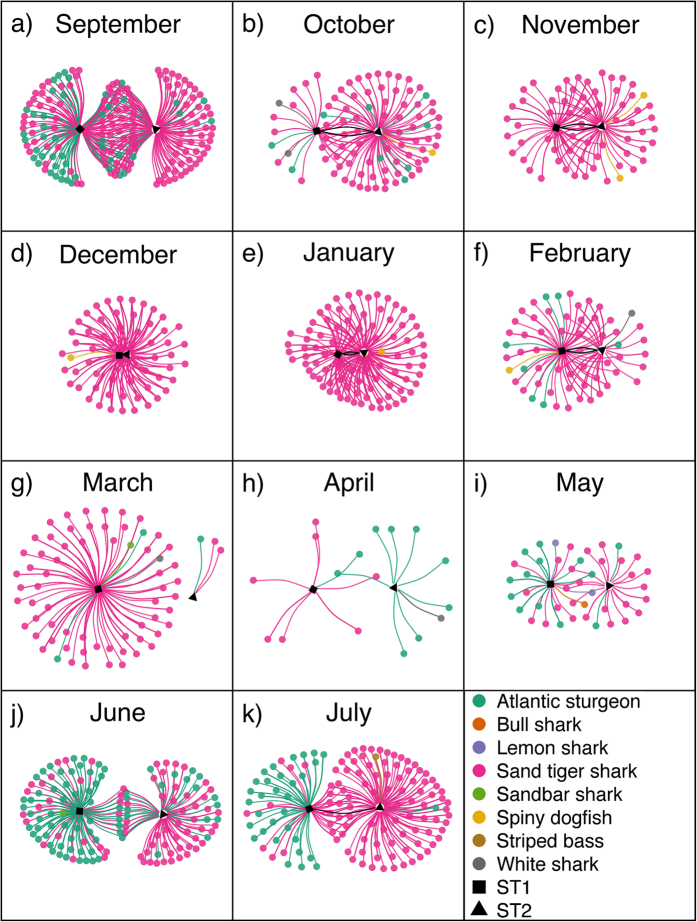
Heterospecific network graphs showing individuals encountered by sand tigers *Carcharias taurus* ST1 and ST2 from September 2012 to July 2013. Points connected only to ST1 (■) were only encountered by ST1 during each month. Points connected only to ST2 (▲) were only encountered by ST2 during each month. Points in the center were encountered by both ST1 and ST2 at some point during the month. Black lines connecting ST1 and ST2 represent co-encounters between ST1 and ST2.

**Figure 3:**
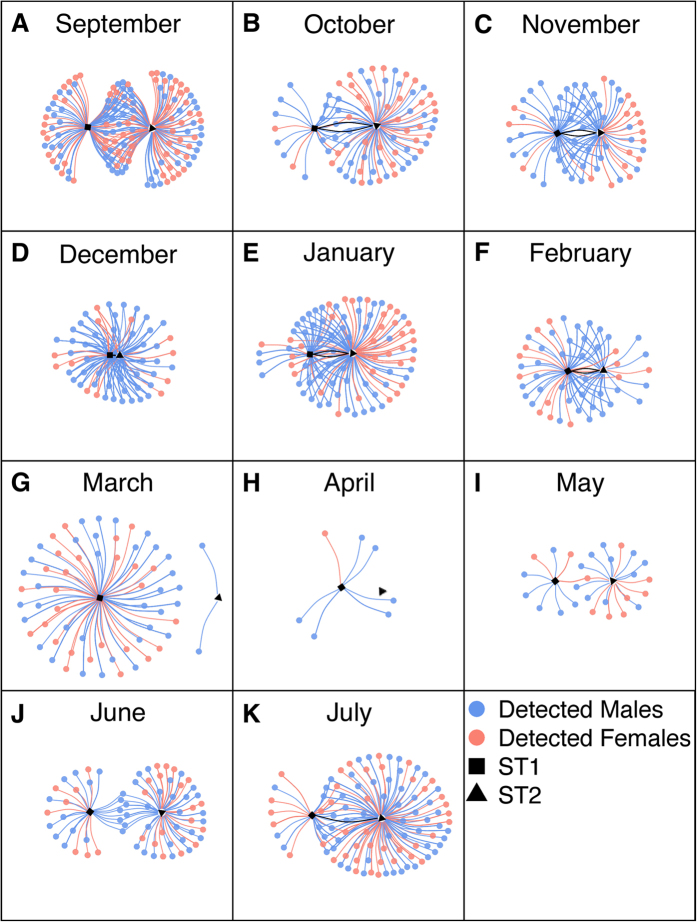
Network graphs of sand tigers *Carcharias taurus* encountered by ST1 and ST2 from September 2012 to July 2013. Points connected only to ST1 (■) were only encountered by ST1 during each month. Points connected only to ST2 (▲) were only encountered by ST2 during each month. Points in the center were encountered by both ST1 and ST2 at some point during the month. Black lines connecting ST1 and ST2 represent co-encounters between ST1 and ST2.

**Figure 4:**
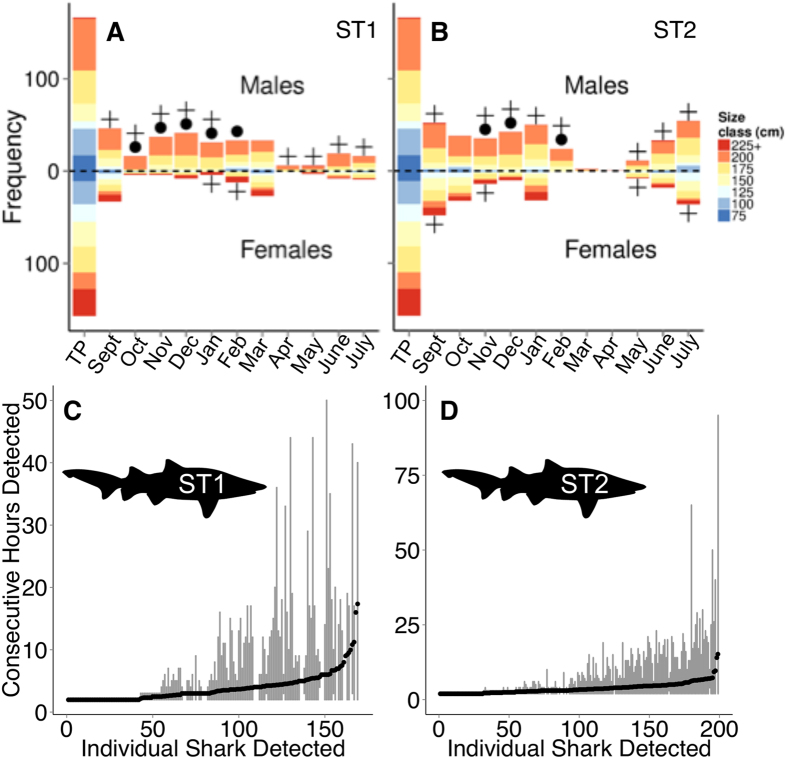
Frequency of male and female sand tigers Carcharias taurus encountered by (**A**) ST1 and (**B**) ST2 between September 2012 and July 2013. Size classes for sand tigers encountered are fork lengths rounded to the nearest 25 cm bin between 75–225+ cm. Size and sex distribution of tagged population (TP) represents the size class and sex at time of tagging of all sand tigers in the Atlantic Cooperative Telemetry (ACT) Network potentially carrying acoustic tags during this study. Symbols represent significant differences between the M:F ratio (●), and the ratio of sand tigers in each size class (+) of encountered sand tigers and sand tigers carrying tags in the ACT Network. All sand tigers were detected for at least two consecutive hours. The mean (black points) and range (grey vertical bars) consecutive hours detected throughout the year for individual sand tigers detected by (**C**) ST1 and (**D**) ST2 are displayed.

For the month of September, ST1 and ST2 were summering in Delaware Bay and the surrounding coastal waters (Fig. 1). During this time they were associated with other individual sand tigers and with Atlantic sturgeon *Acipenser oxyrhynchus oxyrhynchus* (Fig. 2a). During this time, fusion in the inter- and intraspecific communities is suggested by the high degree of overlap in the network graphs (Figs 2a and 3a). The diversity (individual richness and evenness) of sand tigers encountered by both individuals was relatively high throughout the month (Supplemental Fig. 3). The strong network overlap between ST1 and ST2, and their high encounter rate with many different individual sand tigers (Fig. 4A,B), suggests that Delaware Bay provides abundant resources during summer foraging. Sand tigers commonly feed on Atlantic menhaden *Brevoortia tyrannus*^27^, which are found in high abundance in Delaware Bay and the Mid-Atlantic Bight^28^. Reduced competition for food has been shown to support larger group sizes and fusion behavior in primates^9^,^29^,^30^, as well as herding animals^6^.

### South Migration

As ST1 and ST2 left the Delaware coastal ocean on their southern migration in October and November (Fig. 1), we observed fission of the sand tiger group. The sand tigers encountered shifted from well a mixed group in terms of size and sex, to a group of almost exclusively males of a similar size range (~175–200 cm) (Figs 3B,C and 4A,B), which was significantly different than the tagged population of sand tigers (Supplemental Table 1). ST1 and ST2 also encountered fewer Atlantic sturgeon, however, encounters with telemetered white sharks *Carcharodon carcharias* and spiny dogfish *Squalus acanthias* were documented (Fig. 2b,c).

Sexual segregation in sand tigers during the fall migration along the east coast of the United States has been documented^25^, with the majority of mature females moving offshore to shelf waters, and males migrating south along the coast. The shift from a mixture of males and females to almost exclusively males around ST1 and ST2 during their southern migration supports these findings (Figs 3B,C and 4A,B). Sexual segregation is common in elasmobranch species^18^,^31^,^32^, and appears to be occurring with sand tigers as well^25^,^33^. For some mammals (elephants^34^, dolphins^35^, moose^36^, kangaroos^37^), males form loose-knit groups to share mating, foraging or navigational knowledge, maintain access to sparring partners, or defend against predators and interspecific competition^34^. It is possible that male sand tigers are displaying similar male-group behavior during times of migration^25^,^38^,^39^. Male grouping in sharks may also be due to avoidance of males by females, to reduce mating injury and pressure^40^. The highest sand tiger group similarity throughout their entire migration was from November - February (Supplemental Fig. 2A,B), indicating that ST1 and ST2 were re-encountering individual sand tigers from one month to the next. Further work is needed to quantify the fine-scale association patterns within these loose-knit sand tiger aggregations to fully explain this behavior.

### Community Bottleneck

The group of sand tigers associated with ST1 and ST2 again fuses in March and December respectively (Fig. 3D–G). This fusion event likely occurs around the Carolinas based on the location records of ST1 and ST2 (Fig. 1), and anecdotal evidence from researchers whose telemetered fishes (sandbar sharks *Carcharhinus plumbeus*, spiny dogfish) were detected during this time (Fig. 2d–g). ST1 and ST2 mainly encountered similar sand tigers during this community bottleneck phase (up to 80% similar detections in December; Supplemental Fig. 5), but Atlantic sturgeon, white sharks and spiny dogfish were also detected (Fig. 2d–g). ST1 and ST2 encountered each other occasionally in October-January, but peak co-encounters occurred during the middle of this community bottleneck stage in February (Supplemental Fig. 4). The community bottleneck (Dec - Mar) appears related to the sudden increase in overall detections of conspecifics, and notably the reappearance of females (Figs 3D–G and 4A,B). By January the sex ratio of the sand tiger population encountered by ST2 was not significantly different than the tagged population (Fig. 4B, Supplemental Table S1). In March, the size and sex ratio of sand tigers encountered by ST1 reflected that of the tagged population (Fig. 4A, Supplemental Table 1).

The community bottleneck event is likely a reflection of habitat constriction, as well as attraction of animals to physical structure in this region, similar to fish aggregating at seamounts^41^ or around prey resources^42^. Sand tiger sharks seem to prefer water less than 200 m in depth^43^, therefore we suggest that the community bottleneck observed may be driven by the narrowing of the continental shelf near Cape Hatteras, North Carolina (Fig. 1). In addition, sand tigers and other species detected may have been attracted to the multitude of shipwrecks and artificial reefs in the “Graveyard of the Atlantic” between Cape Hatteras, NC, and Cape Lookout, NC (Fig. 1)^44^. Aggregations of sand tigers on these wrecks attract a sizeable dive tourism industry in this area. These shipwrecks may serve as fixed locations to assist in migration, or points where sand tigers and other predators aggregate to take advantage of a food resource (prey fish are also attracted to the structure of the wrecks)^44^, or possibly to look for mates (suggested by reoccurrence of the large females in the detection records).

### Dispersal

As early as March, the network graph for ST2 reveals a second group fission event, which is also reflected in the network graphs of ST1 by April (Fig. 3G,H). There are very few sand tiger encounters for both ST1 and ST2 in April and May, suggesting the group of sand tigers that had fused around the bottleneck at Cape Hatteras had dispersed (Figs 3H,I and 4A,B). While ST1 and ST2 were encountering fewer sand tigers, they did encounter multiple heterospecifcs (Fig. 2g–i). ST1 encountered a variety of elasmobranchs (bull shark *Carcharhinus leucas*, lemon shark *Negaprion brevirostris*, sandbar shark, white shark), in addition to sand tigers and Atlantic sturgeon (Fig. 2g–i). This is supported by the community similarity analysis, which showed interspecific encounters during this dispersal phase to be markedly dissimilar to those found during the rest of the year (Supplemental Fig. 2C,D). There are no documented location records for ST2 south of Cape Hatteras, and between March and May, ST2 went for several weeks without detecting any other telemetered sand tiger, showing a complete dissolution of the sand tiger aggregations previously recorded. This may suggest that the benefits of group living no longer outweighed the potential benefits of group-dispersal, and the male sand tigers in our study entered a solitary phase, thus reducing intraspecific competition for resources (food, mates, habitat, etc.). Fission of groups is a common adaptation to reduce competition for resources and has been observed in primates^3^ and cetaceans^4^,^5^. However, the time scale of dispersal is often observed daily in contrast to the monthly scale dispersal event observed. It is possible that ST1 and ST2 shifted from aggregations of telemetered sand tigers, to aggregations of sand tigers not carrying transmitters, thus accounting for the absence of detections. However, given ST1 and ST2’s proximity to other telemetered sand tigers throughout the rest of the year, this is unlikely, and it is still apparent that ST1 and ST2 moved away from the sand tigers it was previously encountering.

### North Migration

The northern migration of ST1 and ST2 appeared to occur faster than the southern migration, with both sand tigers traveling from the Carolinas to the Mid-Atlantic coastal ocean and Delaware Bay between May and June (Fig. 1). During their northern migration and especially once they reached the Maryland and Delaware coastal ocean, fusion of sand tigers again occurred (Fig. 3I,J), as evidenced by increased diversity in the sex and size of encountered sand tigers (Fig. 4A,B). However, the interspecific network graphs suggest that ST1 and ST2 chose different migratory routes, given that ST1 encountered many Atlantic sturgeon, while ST2 encountered more conspecifics (Fig. 2i,j). ST1 did not re-encounter ST2 until they had both returned to their Delaware Bay summering grounds in mid-July (Fig. 3K), after four months of separation, where the population of sand tigers encountered by ST1 and ST2 again became representative of the tagged population in terms of size distribution and sex ratio (Figs 3J,K and 4, Supplemental Table S1).

### Sand Tiger Association

While it is difficult to understand to what degree ST1 and ST2 were associating with the telemetered sand tigers they encountered, and whether or not it was external or internal forcings driving the group dynamics, we believe that the length of time that some sand tigers spent together is notable. ST1 and ST2 encountered other sand tigers for an average of 33 (range 2–294 hr) and 44 cumulative hours (range 2–335 hr) throughout the study. The top 10% of sand tigers encountered by ST1 and ST2 were encountered for 93–294 (n = 17) and 117–335 (n = 20) cumulative hours, and were almost all male sand tigers (ST1: 16 males, 1 female; ST2: 18 males, 2 females). Even more noteworthy is the length of consecutive hours that some sand tigers remained within the detection range of ST1 and ST2 (Fig. 4C,D). While the average length of consecutive hours ST1 and ST2 spent with other sand tigers was only 4 hr, consecutive hour events ranged from 2–50 hr for ST1 and 2–95 hr for ST2 (Fig. 4C,D). The top 1% of consecutive hour events ranged from 20–50 hr (n = 13) and 21–95 hr (n = 20) for ST1 and ST2 respectively, and were predominantly between males (ST1: 10 males, 2 females; ST2: 12 males, 4 female) (Fig. 4C,D). Interestingly, the top 1% consecutive hour detection events occurred in either September-October while ST1 and ST2 were migrating south, or during the community bottleneck in January and March, which is where ST1 and ST2 spent long periods of time with a few large females (FL: 185–246 cm). This suggests that some associations within the population are not random events, and some behavioral choice may be occurring. It is also important to note that code collision, or changes in the detection range of the internally implanted VMTs may inhibit detections, which may cause close associations between sand tigers to be underrepresented. We also note that with a potential detection range of around 400 m, some of our detections may not be due to direct social interactions.

## Conclusions

Vulnerable species that exhibit fission-fusion group dynamics pose a particularly challenging issue for conservation and management because changes in group size and composition can affect population estimates and amplify anthropogenic impacts. For example, when group size and composition (in terms of individual size class and sex) changes throughout time and space, population monitoring efforts may sample populations disproportionate of their true distributions, or anthropogenic perturbations may disproportionately effect one segment of a population (e.g. mature females only). Using only two acoustic transceivers, we were able to monitor inter- and intraspecific associations between hundreds of individuals in the ocean throughout a year. We also have provided strong evidence for fission-fusion behaviors, a complex group dynamic, in a free-living shark species. These behaviors are typically associated with higher order mammalian species, suggesting that further research is needed to explore the behavioral patterns in this, and other species of sharks that tend to form groups.

## Methods

### Tag implantation and recovery

During August and September of 2012, we implanted 20 VEMCO Mobile Transceivers (VMTs, VEMCO Ltd. Nova Scotia, Canada) into sexually mature sand tigers *Carcharias taurus* caught on long-lines in the Delaware Bay, Delaware USA^22^. Of the 20 sand tigers implanted with VMTs, two male sand tigers were successfully recaptured and their implanted VMTs recovered in July and August of 2013, after approximately one year at liberty (336 and 352 days, respectively)^22^. The detection record from the VMT in ST1 was downloaded using a VMT Optical Reader (VEMCO Ltd.). The VMT in ST2 was recovered after the battery had expired, but the data were recovered by VEMCO Ltd. from the non-volatile flash memory. All sand tigers were caught, handled, and released in accordance with guidelines provided by the Delaware Department of Natural Resources and Environmental Control (DNREC; 2012-021F), the Massachusetts Division of Marine Fisheries. All fishing and tagging protocols were approved by the University of Delaware IACUC (1259-2014-0), and the University of Massachusetts Dartmouth IACUC (10-01).

The data recovered from the VMTs included the detection date, time, and the unique tag code detected. Using the database of VEMCO transmitters deployed in the Atlantic Ocean maintained by the Atlantic Cooperative Telemetry (ACT) Network, we matched the tag code to the tag owners and species carrying the detected tag. Tag owners were contacted and asked for permission to use detections of their telemetered fish in this analysis. In return, the time of detection of the detected fish were provided to the respective tag owner. Detections from researchers that did not wish to participate were removed from the dataset (4 detections of 1 transmitter from 1 tag owner), but these detections did not represent novel species or a large portion of the detection record. In addition, detections of tags that could not be identified were removed from the dataset (1988 detections from 13 tags). One tag owner informed us that the sand tiger carrying an acoustic tag encountered by ST1 and ST2 was part of a surf-fishing mortality study and was likely dead. Detections of this tag code were removed because we could not discern whether the tag was still attached to the sand tiger, or had been shed when the sand tiger suffered mortality. To add additional context to the detections of conspecifics, metadata (sex, fork length, total length) collected from each sand tiger at the time of tagging were matched to the detection record of all detected sand tigers. Using these data, we were able to examine how changes in the sizes and sex of sand tigers encountered by ST1 and ST2 drove the changes in the similarity and diversity analyses (see below).

### Geographic location of detection events

We attempted to use Pop-up Satellite Archival Tags (PSATs, Sea-tag MOD, Desert Star Llc, California, USA) to estimate the locations of the sand tigers carrying VMTs throughout their year at liberty. However, due to malfunction, shedding, or other limitations, we were unable to reconstruct the seasonal paths for any of the tagged animals using the PSAT data. We were, however, able to use the pop-up locations of the PSATS on ST1 and ST2, as well as the pop-up locations from other sharks detected by the VMTs in ST1 and ST2 within seven days of the pop-up event, to get a few estimates of where these sharks were located. To augment our position estimates of these sharks, we used the moored acoustic receiver arrays maintained by members of the ACT Network to provide location records for ST1 and ST2 between the time of tagging and their recapture. In addition, most researchers whose tags were detected by ST1 or ST2 provided acoustic detections of their telemetered fish that occurred within seven days (before or after) of the detection event on the VMTs in ST1 and ST2.

For each detection event (a detection of a tagged fish by the VMTs in ST1 or ST2), we attempted to match a location to the event using the acoustic detections on moored receivers, the VMTs and the PSAT pop-up locations. Locations could be primary locations (location records of ST1 and ST2 on the day of the detection event), secondary (locations records of ST1 and ST2 within seven days of the detection event), or tertiary (location records of the telemetered fish detected by ST1 and ST2 within seven days of the detection event). However, we were unable to determine the location, or approximate location for every detection event using these methods.

### Community structure, behavior, and social network analyses

Leveraging the efforts of researchers in the ACT Network, we computed diversity indices and performed social network analyses to describe temporal changes in the inter- and intraspecific community associated with ST1 and ST2. Interspecific detection totals were normalized using the total number of active tags available in the ACT Network during our study, which accounts for species with differing levels of tagging effort. The Bray-Curtis measure of community dissimilarity was calculated using the normalized abundance of unique species detected each month, as well as the abundance of unique sand tigers detected each month, with the vegdist (*vegan*^45^) function in R^46^. This measure was converted to community similarity for easier interpretation by subtracting the Bray-Curtis dissimilarity indices from one.

Using only the detections of conspecifics over time, we computed the species accumulation rarefaction curve using the specaccum (*vegan*^45^) function in R. In this case, the curve depicts the mean accumulation of unique sand tigers encountered and its confidence intervals. The average species accumulation curves begin to plateau after 11 months, indicating by the end of our study new sand tigers were not being detected frequently. This suggests comparisons of diversity indices and community structure changes within the sand tiger population encountered by ST1 and ST2 over time were appropriate, as the population was likely well sampled.

We calculated Shannon’s diversity index (H) of unique sand tigers detected by ST1 and ST2 each month using the diversity (*vegan*^45^) function in R. The Shannon’s index takes into account richness, which, in this case, includes unique sand tigers encountered by ST1 and ST2 throughout each month, as well as the evenness of the number of days in each month that unique sand tigers were encountered. To add context to the changes in sand tiger diversity encountered by ST1 and ST2 among months, we plotted the number of sand tigers detected each month classified by sex and size. We then used a chi-square goodness-of-fit test (chisq.test{*stats*}^46^) in R to test for significant differences in the sex ratio, and the distribution of sand tigers in 25 cm size bins from 75–225+ cm bins, between the available tagged population of sand tigers and the observed distributions encountered by ST1 and ST2 each month. Monte Carlo simulation was used to calculate the p-value when comparing size distributions because some expected frequencies were too small (<5), resulting in low power in the chi-square approximation.

To explore in more detail at what level the sand tigers encountered by ST1 and ST2 may have been associating, we queried the detection record for cumulative hours when each individual sand tiger was detected by ST1 and ST2. This allowed us to identify individual sand tigers that were detected more often than the rest of the sand tigers encountered by ST1 and ST2. In addition, the lengths of consecutive hours that ST1 and ST2 were associated with individual sand tigers was calculated using the rle function in R.

For context, the proportion of telemetered sand tigers that were encountered by both ST1 and ST2 out of all of the sand tigers encountered by ST1 and ST2 during that month was plotted, along with the number of co-encounters between ST1 and ST2 per month. Finally, social network graphs were plotted displaying the individual sand tigers colored by sex, and the individuals of each species detected each month ST1 and ST2 carried VMTs using the *igraph*^47^ package in R.

## Additional Information

**How to cite this article**: Haulsee, D. E. *et al*. Social Network Analysis Reveals Potential Fission-Fusion Behavior in a Shark. *Sci. Rep*. **6**, 34087; doi: 10.1038/srep34087 (2016).

## Supplementary Material

Supplementary Information

## Figures and Tables

**Table 1:** Detection record summary from archival VEMCO Mobile Transceivers implanted in ST1 and ST2.

Common Name	Scientific Name	Detected by ST1	Detected by ST2	Active Tags (ACT)
Atlantic Sturgeon	*Acipenser oxyrhynchus oxyrhynchus*	120	57	1142
Bull Shark	*Carcharhinus leucas*	1	0	31
Lemon Shark	*Negaprion brevirostris*	2	0	173
Sand Tiger	*Carcharias taurus*	170	200	325
Sandbar Shark	*Carcharhinus plumbeus*	2	0	23
Spiny Dogfish	*Squalus acanthias*	1	2	132
Striped Bass	*Morone saxatilis*	0	1	513
White Shark	*Carcharodon carcharias*	2	2	29

Active Tags are acoustic transmitters deployed by researchers associated with the Atlantic Cooperative Telemetry (ACT) Network that were potentially active during this study (24 Aug 2012 to 27 Jul 2013).

## References

[b1] Jacoby D. M. P., Croft D. P. & Sims D. W. Social behaviour in sharks and rays: analysis, patterns and implications for conservation. Fish Fish 13, 399–417 (2012).

[b2] Couzin I. D. Behavioral ecology: social organization in fission fusion societies. Curr Biol 16, R169–R171 (2006). 16527735 10.1016/j.cub.2006.02.042

[b3] Chapman C. A., Chapman L. J. & Wrangham R. W. Ecological constraints on group size: an analysis of spider monkey and chimpanzee subgroups. Behav Ecology Sociobiol 36, 59–70 (1995).

[b4] Parra G. J., Corkeron P. J. & Arnold P. Grouping and fission–fusion dynamics in Australian snubfin and Indo-Pacific humpback dolphins. Anim Behav 82, 1423–1433 (2011).

[b5] Pearson H. C. Influences on dusky dolphin (*Lagenorhynchus obscurus*) fission-fusion dynamics in Admiralty Bay, New Zealand. Behav Ecology Sociobiol 63, 1437–1446 (2009).

[b6] Fortin D. . Group-size-mediated habitat selection and group fusion-fission dynamics of bison under predation risk. Ecology 90, 2480–2490 (2009). 19769126 10.1890/08-0345.1

[b7] Kelley J. L., Morrell L. J., Inskip C., Krause J. & Croft D. P. Predation risk shapes social networks in fission-fusion populations. PloS one 6, e24280 (2011). 21912627 10.1371/journal.pone.0024280 PMC3166168

[b8] Wallace R. B. Towing the party line: territoriality, risky boundaries and male group size in spider monkey fission–fusion societies. Am J Primatol 70, 271–281 (2008). 17894384 10.1002/ajp.20484

[b9] Aureli F. . Fission‐Fusion Dynamics: New Research Frameworks. Curr Anthropol 49, 627–654 (2008).

[b10] van Schaik C. P. The socioecology of fission-fusion sociality in Orangutans. Primates 40, 69–86 (1999). 23179533 10.1007/BF02557703

[b11] Kerth G., Ebert C. & Schmidtke C. Group decision making in fission-fusion societies: evidence from two-field experiments in Bechstein’s bats. P Roy Soc B-Biol Sci 273, 2785–2790 (2006). 10.1098/rspb.2006.3647 PMC1635504 17015328

[b12] Kerth G., Perony N. & Schweitzer F. Bats are able to maintain long-term social relationships despite the high fission-fusion dynamics of their groups. P Roy Soc B-Biol Sci 278, 2761–2767 (2011). 10.1098/rspb.2010.2718 PMC3145188 21307051

[b13] Leu S. T., Bashford J., Kappeler P. M. & Bull C. M. Association networks reveal social organization in the sleepy lizard. Anim Behav 79, 217–225 (2010).

[b14] While G. M., Uller T. & Wapstra E. Family conflict and the evolution of sociality in reptiles. Behav Ecol 20, 245–250 (2009).

[b15] Croft D. P., Krause J. & James R. Social networks in the guppy (Poecilia reticulata). P Roy Soc B-Biol Sci 271, S516–S519 (2004). 10.1098/rsbl.2004.0206 PMC1810091 15801620

[b16] Northcutt R. G. Elasmobranch central nervous system organization and its possible evolutionary significance. Am Zool 17, 411–429 (1977).

[b17] Connor R. C., Mann J., Tyack P. L. & Whitehead H. Social evolution in toothed whales. Trends Ecol Evol 13, 228–232 (1998). 21238276 10.1016/s0169-5347(98)01326-3

[b18] Jacoby D. M. P., Busawon D. S. & Sims D. W. Sex and social networking: the influence of male presence on social structure of female shark groups. Behav Ecol 21, 808–818 (2010).

[b19] Coles R. J. Notes on the sharks and rays of Cape Lookout, N. P Biol Soc Wash 28, 89–94 (1915).

[b20] Würsig B. In Dolphin cognition and behavior: A comparative approach Shusterman R. J., Thomas J. A., Wood F. G. , Eds. (Lawrence Erlbaum, New Jersey, 1986), pp. 347–369.

[b21] Pollard D. & Smith A. “*Carcharias taurus”*. Version 2012.2: IUCN Red List of Threatened Species (2009).

[b22] Haulsee D. E., Fox D. A., Breece M. W., Clauss T. M. & Oliver M. J. Implantation and Recovery of Long-Term Archival Transceivers in a Migratory Shark with High Site Fidelity. PloS one 11, e0148617 (2016). 26849043 10.1371/journal.pone.0148617 PMC4744049

[b23] Goldman K. J., Branstetter S. & Musick J. A. A re-examination of the age and growth of sand tiger sharks, *Carcharias taurus*, in the western North Atlantic: the importance of ageing protocols and use of multiple back-calculation techniques. Environ Biol Fish 77, 241–252 (2006).

[b24] Kneebone J., Chisholm J. & Skomal G. Movement patterns of juvenile sand tigers (Carcharias taurus) along the east coast of the USA. Mar Biol 161, 1149–1163 (2014).

[b25] Teter S. M. . Migratory patterns and habitat use of the sand tiger shark (*Carcharias taurus*) in the western North Atlantic. Mar Fresh Res 66, 158 (2015).

[b26] Baker L. L. . Probability of detecting marine predator-prey and species interactions using novel hybrid acoustic transmitter-receiver tags. PloS one 9, e98117 (2014). 24892286 10.1371/journal.pone.0098117 PMC4043729

[b27] Gelsleichter J., Musick J. A. & Nichols S. Food habits of the smooth dogfish, *Mustelus canis*, dusky shark, *Carcharhinus obscurus*, Atlantic sharpnose shark, *Rhizoprionodon terraenovae*, and the sand tiger, *Carcharias taurus*, from the northwest Atlantic Ocean. Environl Biol Fish 54, 205–217 (1999).

[b28] “Atlantic menhaden stock assessment report for public peer review” Atlantic States Marine Fisheries Commission Rep. 99–01 (1999).

[b29] Snaith T. V. & Chapman C. A. Primate group size and interpreting socioecological models: do folivores really play by different rules. Evol Anthropol 16, 94–106 (2007).

[b30] Symington M. M. Food competition and foraging party size in the black spider monkey (*Ateles paniscus* Chamek). Behaviour 105, 117–132 (1988).

[b31] Mucientes G. R., Queiroz N., Sousa L. L., Tarroso P. & Sims D. W. Sexual segregation of pelagic sharks and the potential threat from fisheries. Biol Lett 5, 156–159 (2009). 19324655 10.1098/rsbl.2008.0761 PMC2665836

[b32] Wearmouth V. J. & Sims D. W. Sexual segregation in marine fish, reptiles, birds and mammals: behaviour patterns, mechanisms and conservation implications. Adv Mar Biol 107–170 (2008). 18929064 10.1016/S0065-2881(08)00002-3

[b33] Bissonnette A., Bischofberger N. & van Schaik C. P. Mating skew in Barbary macaque males: the role of female mating synchrony, female behavior, and male-male coalitions. Behav Ecol Sociobiol 65, 167–182 (2011). 22448085 10.1007/s00265-010-1023-z PMC3291840

[b34] Chiyo P. I. . Association patterns of African elephants in all-male groups: the role of age and genetic relatedness. Anim Behav 81, 1093–1099 (2011).

[b35] Lusseau D. . The bottlenose dolphin community of Doubtful Sound features a large proportion of long-lasting associations. Behav Ecol Sociobiol 54, 396–405 (2003).

[b36] Miquelle D. G., Peek J. M. & Van Ballenberghe V. Sexual segregation in Alaskan moose. Wildlife Monogr 3–57 (1992).

[b37] MacFarlane A. M. & Coulson G. Boys will be boys: social affinity among males drives social segregation in western grey kangaroos. J Zool 277, 37–44 (2009).

[b38] Bansemer C. S. & Bennett M. B. Sex- and maturity-based differences in movement and migration patterns of grey nurse shark, *Carcharias taurus*, along the eastern coast of Australia. Mar Fresh Res 62, 596 (2011).

[b39] Otway N. M. & Ellis M. T. Pop-up archival satellite tagging of *Carcharias taurus*: movements and depth/temperature-related use of south-eastern Australian waters. Mar Fresh Res 62, 607 (2011).

[b40] Wearmouth V. J. & Sims D. W. Sexual segregation of marine fish, reptiles, birds and mammals: behaviour patterns, mechanisms and conservation implications. Adv Mar Biol 54, 107–170 (2008). 18929064 10.1016/S0065-2881(08)00002-3

[b41] Holland K. N. & Grubbs R. D. Fish visitors to seamounts: tunas and billfish at seamounts. In: Pitcher T. J. . (eds) Seamounts: ecology, conservation and management. Fish and aquatic resources series, Blackwell Scientific, Oxford 189–201 (2007).

[b42] de la Parra Venegas R. . An unprecedented aggregation of whale sharks, Rhinocodon typus, in Mexican coastal waters of the Caribbean Sea. PLoS One 6, e18994 (2011). 21559508 10.1371/journal.pone.0018994 PMC3084747

[b43] Compagno L. J. V. Sharks of the world: an annotated and illustrated catalogue of shark species known to date (FAO, Rome, 2001).

[b44] Whitfield P. E., Muñoz R. C., Buckell C. A. & Heesemann L. M. Fish and habitat community assessments on North Carolina shipwrecks: potential sites for detecting climate change in the graveyard of the Atlantic. (Marine Sanctuaries Conservation Series ONMS, Silver Spring, MD, 2011), pp. 39.

[b45] Oksanen J. . vegan: Community Ecology Package. R package version 2.2-1, http://CRAN.R-project.org/package=vegan (2015).

[b46] R core team. R: A language and environment for statistical computing. R Foundation for Statistical Computing, Vienna, Austria. http://www.R-project.org/ (2015).

[b47] Csardi G. & Nepusz T. The igraph software package for complex network research, InterJournal, Complex Systems 1695, http://igraph.org (2006).

[b48] Amante C. & Eakins B. W. ETOPO1 1 Arc-Minute Global Relief Model: Procedures, Data Sources and Analysis. NOAA Technical Memorandum NESDIS NGDC-24. National Geophysical Data Center, NOAA. (2009), doi: 10.7289/V5C8276M (accessed: 15 Aug 2015).

