# Supplementary material for: Social Network Analysis Reveals Potential Fission-Fusion Behavior in a Shark
Source: Sci Rep. 2016 Sep 30;6:34087. doi: 10.1038/srep34087 (PMC5043177; doi:10.1038/srep34087)
Supplement: Supplementary Information [file srep34087-s1.doc]

Supplementary Materials for

**Social Network Analysis Reveals Potential Fission-Fusion Behavior in a Shark**

Danielle E. Haulsee*, Dewayne A. Fox, Matthew W. Breece, Lori M. Brown, Jeff Kneebone, Gregory B. Skomal, Matthew J. Oliver

*Corresponding author. E-mail: dhaulsee@udel.edu

**This file includes:**

Supplemental Figs. 1-4

Supplemental Table 1


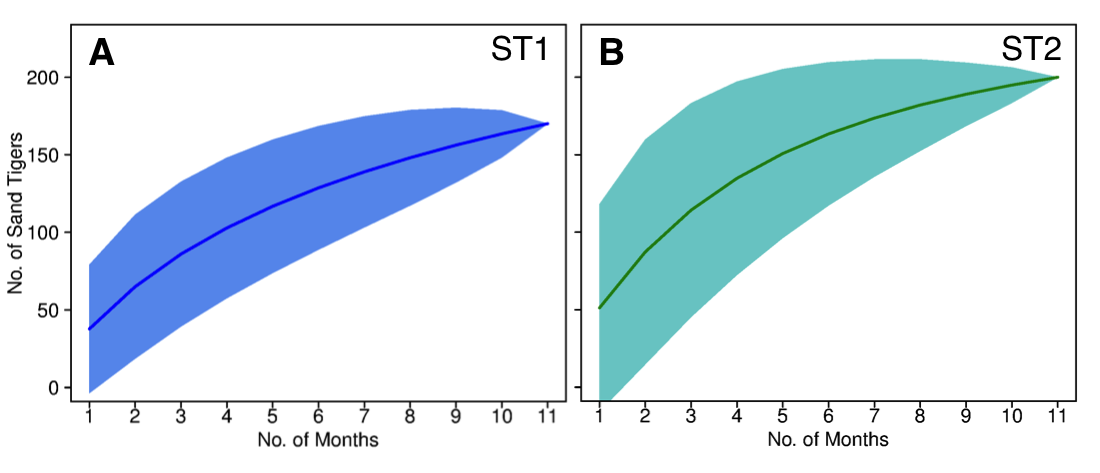
**Supplemental Fig. 1**

Accumulation of individual sand tigers *Carcharias taurus* detected by VEMCO Mobile Transceivers implanted in **A**) ST1 and **B**) ST2 per month. The mean accumulation curve (blue and green solid line) represents the best model for accumulation of new species as new sites (months) are sampled, and is surrounded by the 95% confidence intervals.


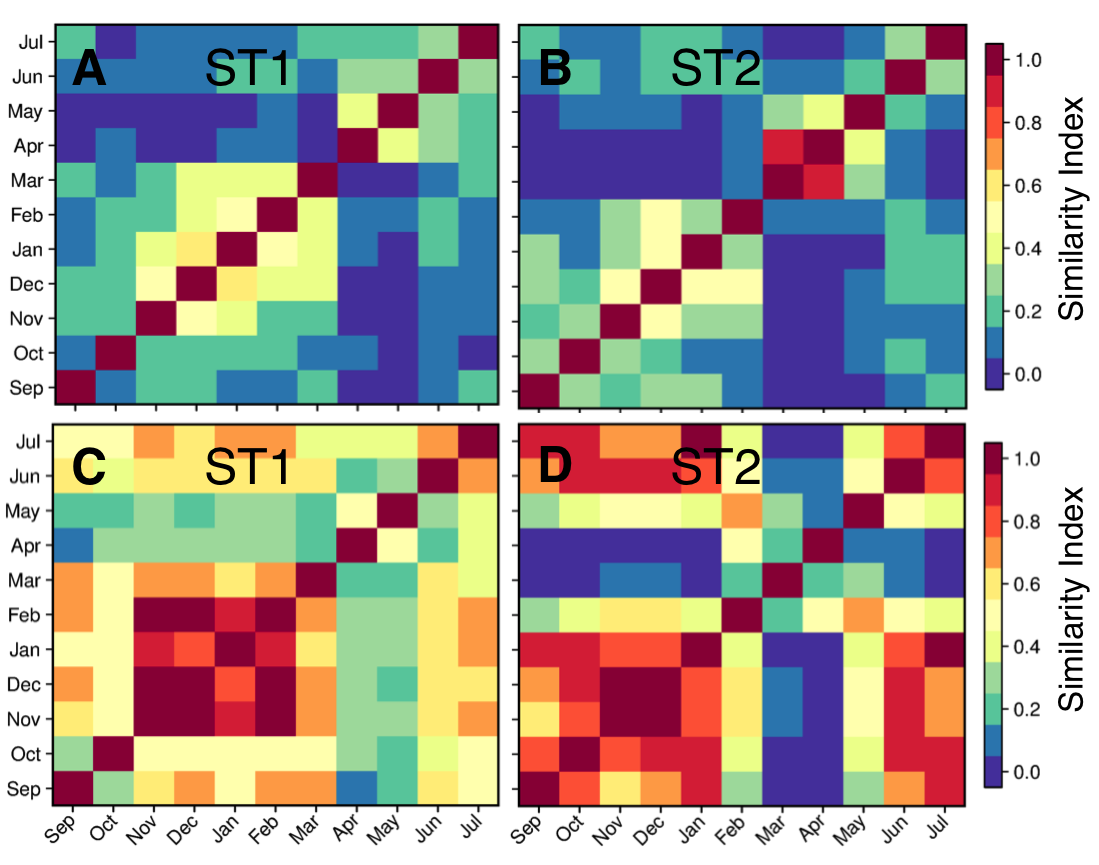
Supplemental Fig. 2

Bray-Curtis Similarity Index for **A-B**) intraspecific and **C-D**) interspecific encounters by sand tigers (*Carcharias taurus*) ST1 and ST2. Warmer colors represent high community similarity between months, and cooler colors represent low community similarity between months.


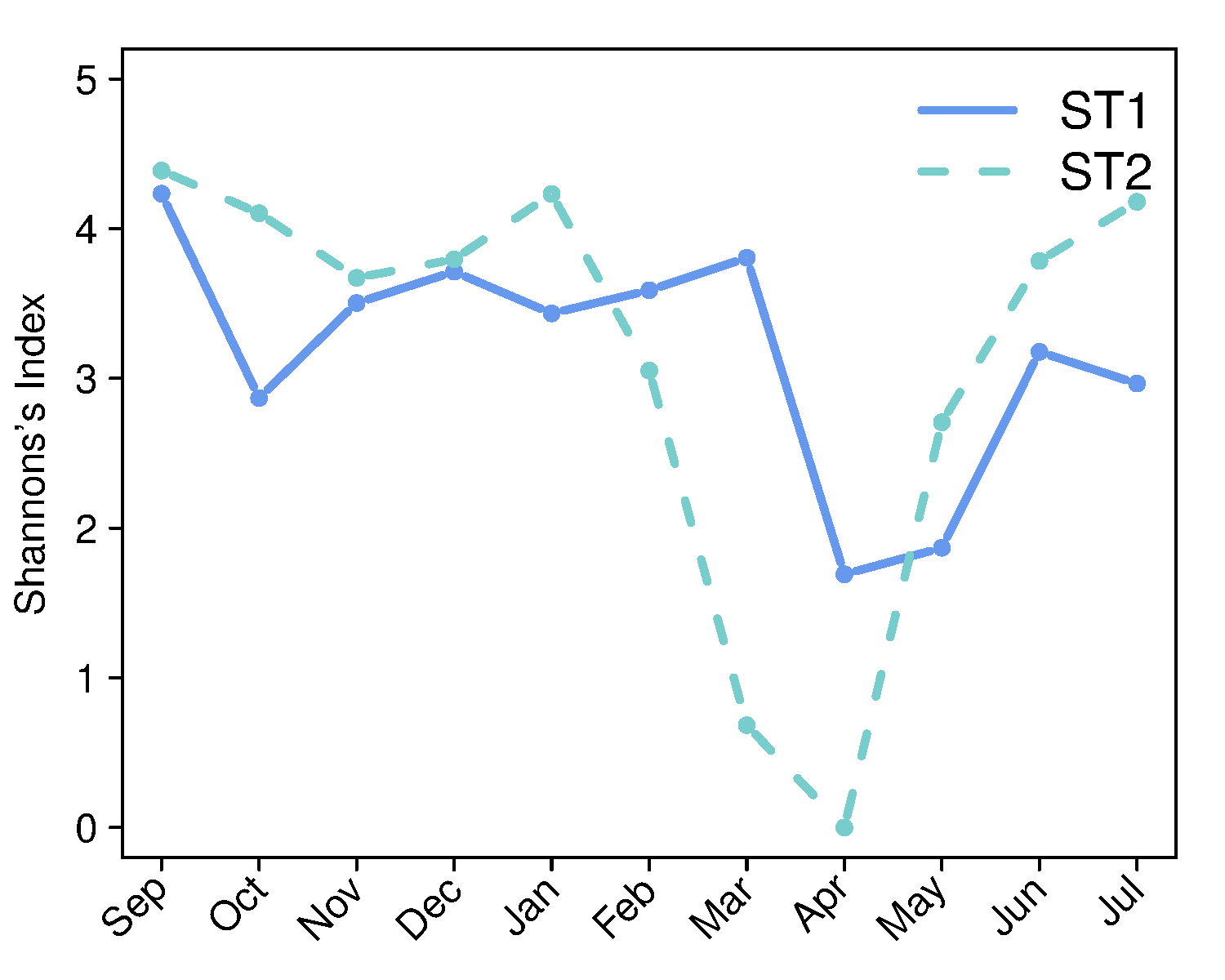
Supplemental Fig. 3

Shannon’s diversity index (H) displaying the diversity of individual sand tigers *Carcharias taurus* encountered by ST1 and ST2 each month.


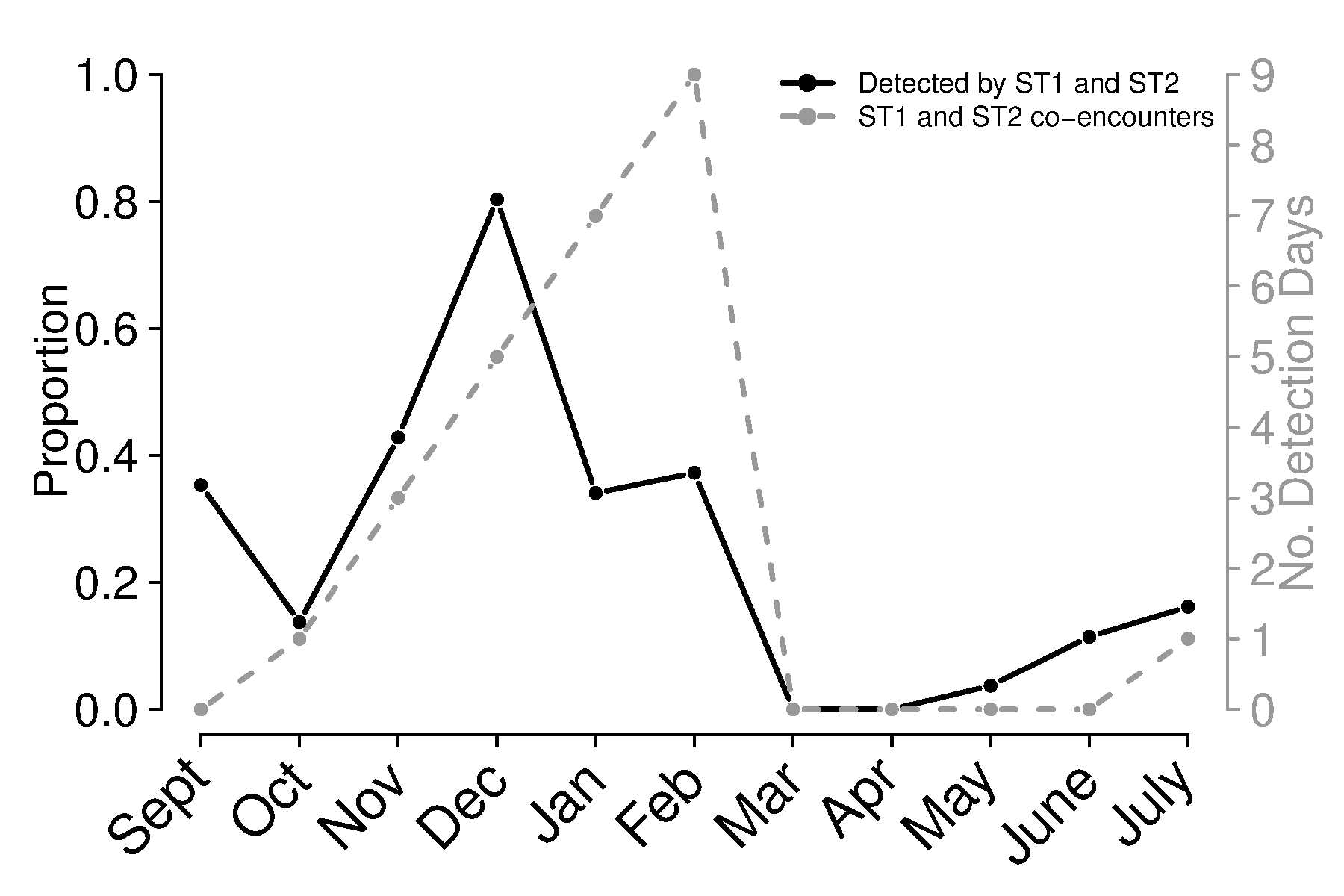


**Supplemental Fig. 4**

Proportion of sand tigers *Carcharias taurus* encountered by either ST1 or ST2 that were encountered by both ST1 and ST2 each month (black line) and the number of days in each month that ST1 and ST2 encountered each other (grey line).

**Supplemental Table 1.**

|  | ST1 | | | | | | ST2 | | | | | |
| --- | --- | --- | --- | --- | --- | --- | --- | --- | --- | --- | --- | --- |
|  | M:F | | Size F | | Size M | | M:F | | Size F | | Size M | |
|  | X^2^ | p | X^2^ | p | X^2^ | p | X^2^ | p | X^2^ | p | X^2^ | p |
| Overall | 3.46 | 0.063 | 12.34 | 0.055 | 20.33 | **0.001** | 0.43 | 0.514 | 16.11 | **0.013** | 17.67 | **0.004** |
| September | 1.35 | 0.245 | 4.14 | 0.661 | 15.39 | **0.010** | 0.02 | 0.875 | 14.15 | **0.027** | 18.27 | **0.004** |
| October | 6.42 | **0.011** | 4.61 | 0.652 | 92.68 | **<0.001** | 0.30 | 0.585 | 8.10 | 0.227 | 10.91 | 0.053 |
| November | 24.40 | **<0.001** | 5.57 | 0.416 | 56.92 | **<0.001** | 7.64 | **0.006** | 13.38 | **0.036** | 48.04 | **<0.001** |
| December | 20.07 | **<0.001** | 5.74 | 0.451 | 168.77 | **<0.001** | 17.61 | **<0.001** | 7.00 | 0.301 | 176.11 | **<0.001** |
| January | 19.06 | **<0.001** | 12.65 | **0.046** | 169.78 | **<0.001** | 3.16 | 0.075 | 12.05 | 0.060 | 17.31 | **0.005** |
| February | 8.44 | **0.004** | 13.66 | **0.033** | 6.68 | 0.236 | 19.65 | **<0.001** | 13.27 | 0.070 | 140.87 | **<0.001** |
| March | 0.40 | 0.527 | 6.02 | 0.419 | 9.36 | 0.094 | 1.87 | 0.503 | NA | NA | 5.74 | 0.255 |
| April | 3.24 | 0.072 | 13.27 | 0.069 | 53.15 | **<0.001** | NA | NA | NA | NA | NA | NA |
| May | 0.81 | 0.369 | 3.79 | 0.696 | 51.92 | **<0.001** | 0.29 | 0.589 | 17.15 | **0.008** | 41.12 | **<0.001** |
| June | 3.77 | 0.052 | 6.92 | 0.317 | 82.16 | **<0.001** | 3.46 | 0.063 | 4.11 | 0.672 | 14.35 | **0.015** |
| July | 1.52 | 0.218 | 8.26 | 0.190 | 32.52 | **<0.001** | 2.49 | 0.115 | 22.33 | **0.001** | 64.13 | **<0.001** |

Chi-squared test results comparing the sex and size ratios of sand tigers *Carcharias taurus* encountered by ST1 and ST2, throughout the study (“Overall”) and for each month, to the expected sex and size ratios from the tagged population of sand tigers in the Atlantic Cooperative Telemetry Network. Significant differences (p < 0.05) are highlighted in bold.
